# Blood-Based Biomarkers for the Preterminal Stage Using Rhesus Macaques Exposed to Acute Total-Body Radiation

**DOI:** 10.3390/ijms27177709

**Published:** 2026-08-28

**Authors:** Matthew W. Brink, Alana D. Carpenter, Rachel C. Mingus, Sarah A. Petrus, Oluseyi O. Fatanmi, Stephen Y. Wise, Vijay K. Singh

**Affiliations:** 1 Division of Radioprotectants, Department of Pharmacology and Molecular Therapeutics, F. Edward Hébert School of Medicine, Uniformed Services University of the Health Sciences, Bethesda, MD 20814, USA; matthew.brink.ctr@usuhs.edu (M.W.B.); alana.carpenter.ctr@usuhs.edu (A.D.C.); rachel.mingus.ctr@usuhs.edu (R.C.M.); sarah.petrus.ctr@usuhs.edu (S.A.P.); oluseyi.fatanmi@usuhs.edu (O.O.F.); stephen.wise.ctr@usuhs.edu (S.Y.W.); 2 Armed Forces Radiobiology Research Institute, Uniformed Services University of the Health Sciences, Bethesda, MD 20814, USA

**Keywords:** preterminal, acute radiation syndrome, rhesus macaques, complete blood count, serum chemistry, biomarkers

## Abstract

Acute radiation syndrome is characterized by progressive hematologic decline and biochemical imbalances, yet the transition from recoverable injury to the preterminal state remains incompletely characterized using routine laboratory assays. Prior proteomic, metabolomic, and transcriptomic studies have revealed biological signatures in preterminal serum and plasma samples from irradiated nonhuman primates, suggesting that the preterminal state represents a distinct phenotype. In this study, complete blood count and serum biochemistry data collected from male and female rhesus macaques (*Macaca mulatta*) exposed to total-body doses of ^60^Co γ-radiation were monitored from seven days prior to irradiation through 60 days post-irradiation. Preterminal samples were collected immediately prior to euthanasia upon determination of moribundity. A series of analyses was performed to characterize differences in injury severity and recovery between survivors and non-survivors. Our findings showed shared early cytopenic decreases in both survivors and non-survivors, consistent with expected radiation-induced hematopoietic injury. Preterminal samples collected between days 14–22 post-irradiation were characterized by erythroid decline and lymphocyte depletion. Most notably, red blood cells, hemoglobin, and hematocrit showed a delayed decline, with significant decreases associated with non-survival. Serum chemistry data was less discriminatory during scheduled sampling, but preterminal comparisons demonstrated significant changes prior to mortality. These results suggest that conventional clinical metrics can help characterize the preterminal phenotype.

## 1. Introduction

The threat of mass-casualty radiation exposure has intensified in recent decades due to nuclear proliferation and potential radiological terrorism, creating an urgent need for improved medical preparedness [1,2]. When such events occur, healthcare systems may face the critical challenge of triaging potentially thousands of victims exposed to ionizing radiation with limited resources and time [3,4]. Current triage approaches rely on early biomarkers to estimate exposure dose and predict survival but lack reliable indicators of imminent death in individuals progressing to the terminal stages of acute radiation syndrome (ARS) [5,6,7]. This gap severely limits clinicians’ ability to make informed decisions about resource allocation and patient care urgency.

Hematopoietic ARS (H-ARS) is the primary life-threatening form of radiation sickness. It occurs following exposure to intermediate doses of ionizing radiation (2–6 Gy) that specifically target the bone marrow. Common side effects include a significant loss of appetite, nausea, and vomiting, which typically emerge during the initial prodromal stage shortly after exposure [8,9,10,11]. As H-ARS progresses to the manifest illness stage, the clinical picture is dominated by pancytopenia, particularly neutropenia and thrombocytopenia. This leads to an increased risk of opportunistic infections and sepsis, as well as hemorrhage characterized by petechiae, ecchymosis, and mucosal bleeding, potentially resulting in death in about 2–8 weeks as a result from radiation-induced anemia or infection-related complications [4,12,13]. The severity and duration of H-ARS are largely dependent on the radiation dose received, with higher doses associated with more profound cytopenia and an increased risk of mortality [10,14,15]. Although H-ARS primarily affects the hematopoietic system, it is also accompanied by alterations in serum chemistry parameters that reflect systemic homeostatic disturbances and dysfunction of multiple organ systems beyond the bone marrow [16].

Identifying the biological signatures of the preterminal state in H-ARS is important for defining the transition from injury onset to irreversible decline. Thus, understanding the physiological changes that occur in the preterminal state is imperative for identifying parameters associated with recovery failure and terminal decline to enable more effective triage, medical intervention, and allocation of resources [13,17]. Recent molecular profiling studies using nonhuman primate (NHP) models have revealed that lethally irradiated animals exhibit distinct biological signatures in samples collected from moribund animals immediately prior to death compared to earlier post-irradiation timepoints [18,19,20,21]. For example, proteomic analyses of preterminal samples demonstrated pronounced dysregulation in pathways related to hemostasis, immune response, and platelet function [20,21]. Similarly, metabolomic studies showed severe disruptions in sphingolipid metabolism, steroid hormone biosynthesis, and glycerophospholipid pathways [18,19]. Gene expression changes have also been characterized in NHP preterminal samples [22], and these results demonstrated that during this stage, the ribosome/transcriptome status remained present (normal range of 18S rRNA values) in ~60% of animals, while overall transcriptomic activity appeared largely silent. The divergence between preserved ribosomal integrity and suppressed global gene expression was shown to serve as an indicator of impending terminal decline.

These molecular alterations in moribund animals immediately prior to death indicate that the transition to moribundity often involves specific pathophysiologic processes that may be detectable through conventional laboratory parameters. Complete blood counts (CBCs) and serum chemistry panels represent widely accessible clinical laboratory measurements that can be performed with standard instrumentation, in contrast to specialized molecular assays such as multi-omics approaches that require sophisticated equipment, methodology, and trained laboratory personnel [23,24,25,26,27]. CBC parameters following ionizing radiation exposure have been extensively characterized and are well-established as biomarkers for radiation injury [5,28,29,30,31,32]. Serum biochemistry data provides additional insight into the overall health of the animal. Therefore, both CBC and serum chemistry parameters have potential utility as accessible biomarkers for identifying individuals at risk of imminent death during radiological mass-casualty scenarios, particularly at lethal radiation doses where rapid triage decisions are vital.

The objective of this study was to characterize CBC and serum chemistry parameters in preterminal samples from rhesus macaques, the preclinical “gold-standard” model for human radiation research [33,34,35,36], exposed to lethal doses of total-body cobalt-60 (^60^Co) γ-radiation. Beyond its significant physiological and genomic relevance to humans, the NHP model has supported the regulatory approval of multiple countermeasures for H-ARS under the FDA Animal Rule [37,38,39,40,41,42]. In this analysis, we compared preterminal samples, collected from moribund animals immediately prior to euthanasia, against both pre-irradiation baselines and various longitudinal post-irradiation timepoints over a 60-day period in an effort to characterize the preterminal phenotype. Notably, early leukocyte and platelet suppression were observed post-irradiation, regardless of survivor status, while decreases in red blood cells (RBCs), hemoglobin (HGB), and hematocrit (HCT) were significantly associated with non-survivors. Serum biochemistry data also showed significant preterminal changes, including increases in blood urea nitrogen (BUN), phosphorus, creatinine, total bilirubin, and alanine aminotransferase (ALT), along with decreases in glucose, total protein, calcium, albumin, and alkaline phosphatase (ALP).

## 2. Results

Through evaluating preterminal CBC and serum chemistry responses following 7.2 and 7.4 Gy TBI, this study characterizes the hematologic and biochemical biomarkers associated with the preterminal state. Animals in both cohorts were monitored for 60 days post-irradiation, and these parameters were analyzed to define the timing of hematologic and biochemical deterioration, evaluate trajectories associated with mortality, and characterize the preterminal state in non-survivors. A Kaplan–Meier curve was generated to evaluate overall survival through study completion to clearly illustrate the time distribution of animal euthanasia (Supplementary Figure S1).

### 2.1. Hematologic Response to TBI

#### 2.1.1. Shared Early Cytopenia and Late Erythroid-Related Divergence Was Observed Between Survivors and Non-Survivors

**Figure 1:**
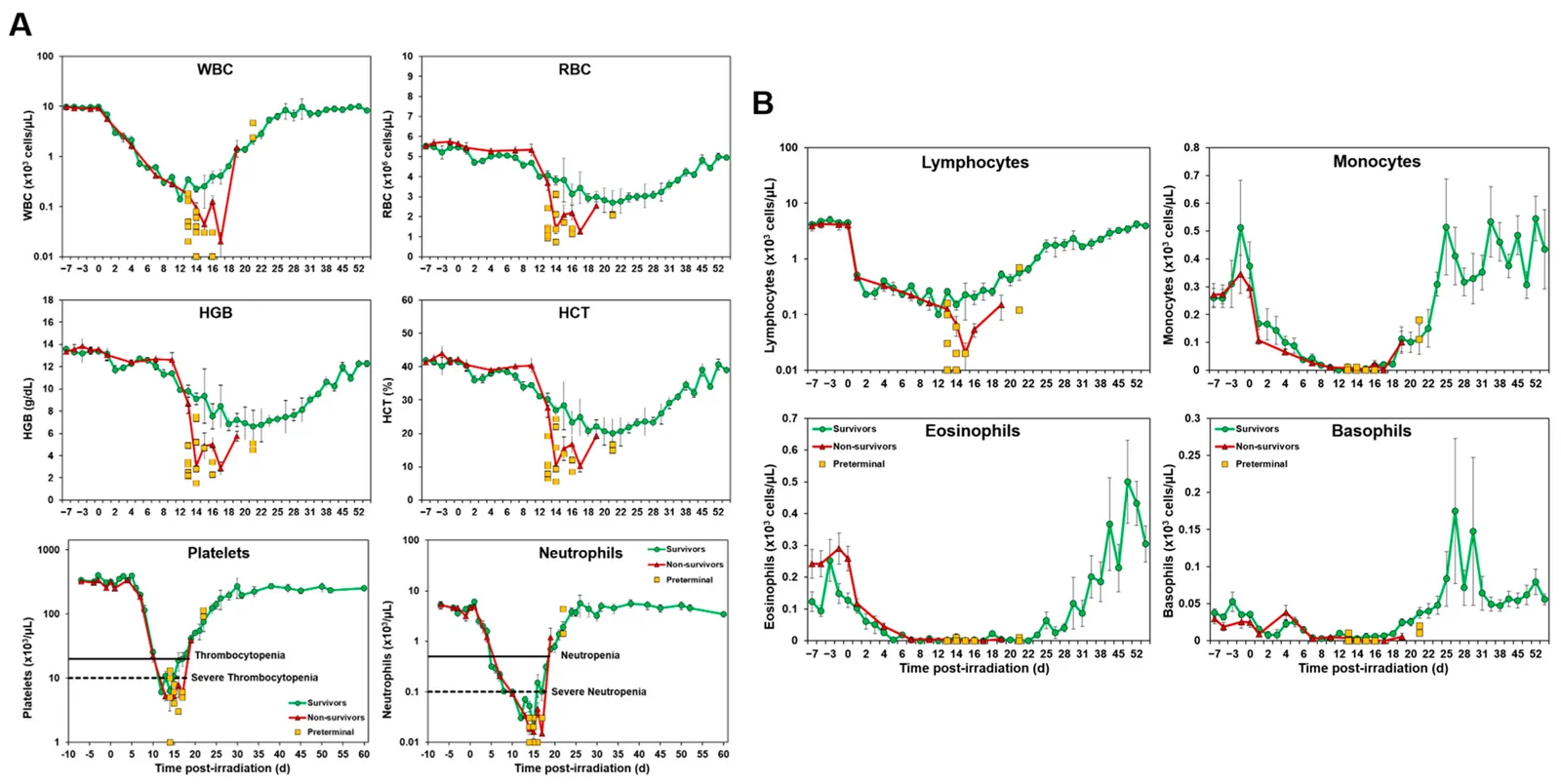
Raw trendlines of various complete blood cell counts (CBCs) stratified by survivors, non-survivors, and preterminal data. These hematologic parameters capture late-stage peripheral blood responses within white blood cells (WBCs), red blood cells (RBCs), hemoglobin (HGB), hematocrit (HCT), platelets, and neutrophils (**A**), and lymphocytes, monocytes, eosinophils, and basophils (**B**), preceding mortality and compare recovery trajectories between outcome groups.

Pooled CBC data from the two cohorts, divided into survivors, non-survivors, and preterminal samples, demonstrate a general decline across all blood cell lineages post-irradiation (Figure 1A,B). However, the timing and rate of this depletion vary considerably, largely reflecting the natural circulating lifespan of each cell type. White blood cells (WBCs) and neutrophils, which mediate immune defense, and platelets, which are critical for hemostasis, declined rapidly within the first 10 days post-irradiation for both survivors and non-survivors. In contrast, RBCs, HGB, and HCT remained relatively stable during the first 10–12 days following TBI before undergoing a more pronounced decline, particularly in non-survivors. This delayed reduction likely reflects the inherently longer lifespan of mature erythrocytes compared with other hematopoietic cell populations. In survivors, all CBC parameters recovered toward baseline by the end of the 60-day study.

Around days 12–14 post-irradiation, a clear separation emerged between non-survivors, preterminal animals, and survivors that successfully underwent hematopoietic recovery. In survivors, recovery of WBCs, platelets, neutrophils, and lymphocytes became evident by days 14–15 post-irradiation. Platelet and neutrophil counts rapidly surpassed the critical thresholds associated with severe thrombocytopenia (<10 × 10^3^ cells/µL) and severe neutropenia (<0.1 × 10^3^ cells/µL), returning to baseline by days 30 and 26, respectively, while lymphocytes exhibited a more gradual and sustained recovery trajectory. An intermediate recovery phase was observed between days 18–20 for monocytes and basophils and was characterized by a rebound that rose substantially above pre-irradiation levels between days 25–45, although values fluctuated throughout this period. A later recovery phase occurred between days 22–25 for RBCs, HGB, HCT, and eosinophils, consistent with the delayed restoration of erythroid and select granulocyte populations following irradiation.

In contrast, most non-survivors did not demonstrate any evidence of recovery after the nadir period. Mortality events and preterminal sample collections clustered within the critical period window between days 14–17 post-irradiation, corresponding directly with when blood cell counts reached their lowest values. Although a sharp increase was observed thereafter, most evidently in WBCs, platelets, and neutrophils on day 20, or the end of the critical period, this apparent rebound was likely influenced by the decreasing sample size of the non-survivors. Notably, two preterminal collections were performed on day 22, at which point these animals had already begun to demonstrate hematological recovery of WBCs, platelets, and neutrophils, but were euthanized following clinical determination of moribundity by the attending veterinarian. The specific moribundity criteria are outlined in detail in the Supplementary Information. Once an animal was determined to be moribund, recovery was considered unlikely given the severity of clinical deterioration. This highlights the highly variable, non-linear nature of radiation injury, where early bone marrow reconstitution can occur asynchronously with overall systemic recovery. Ultimately, it underscores that peripheral hematopoietic recovery can be decoupled from overall survival, demonstrating that a system-specific rebound in blood counts does not guarantee clinical stabilization.

#### 2.1.2. PCA Visualization Supports Separation of the Preterminal Phenotype

**Figure 2:**
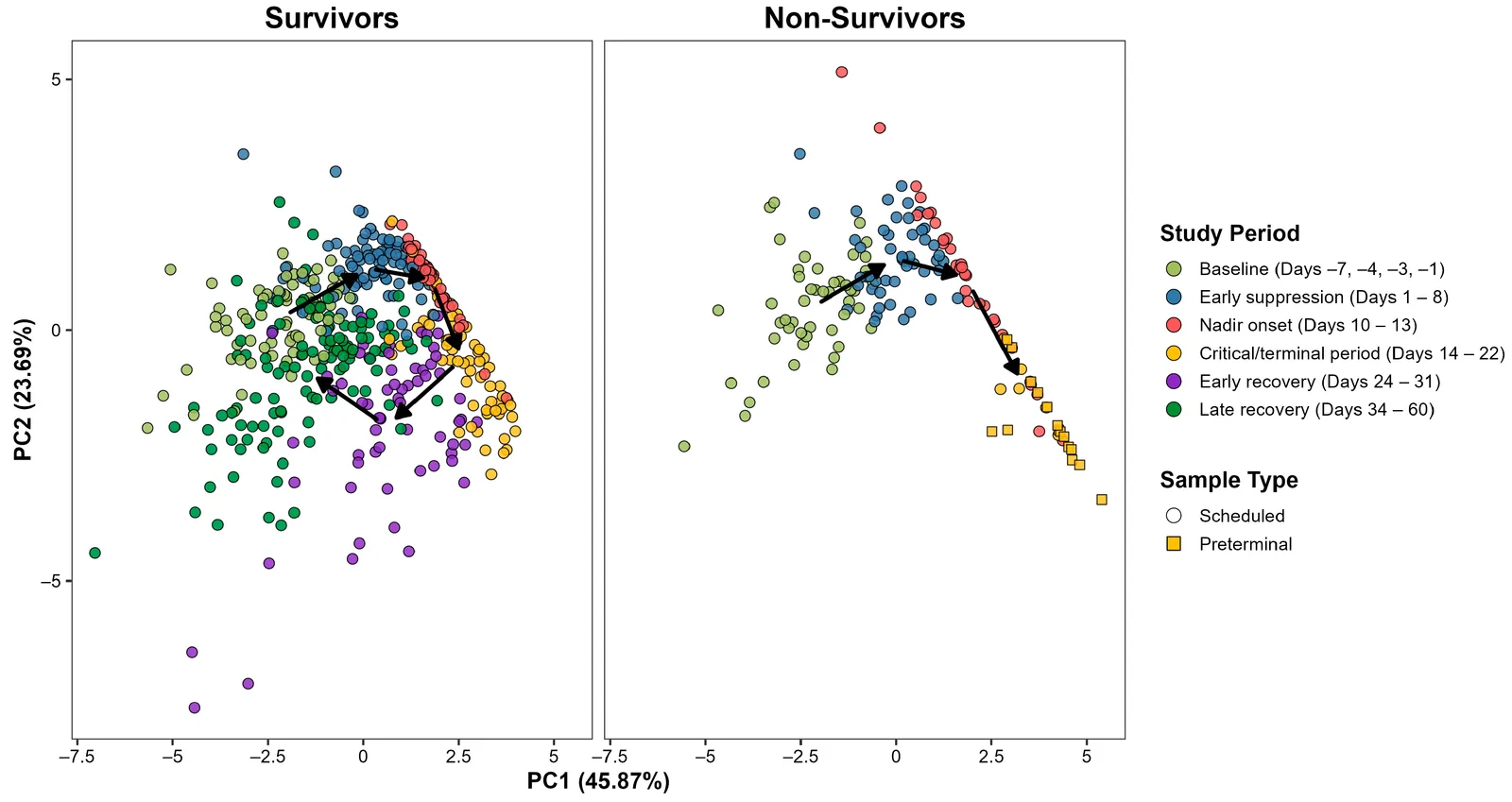
Principal component analysis (PCA) of complete blood cell (CBC) parameters following total-body irradiation (TBI). Parameters were z-scored prior to PCA to ensure each parameter contributed equally regardless of scale. Samples were color-coded into biologically distinct response periods to illustrate hematological response post-irradiation. Arrows are used to connect the centroids of each study period.

Unsupervised principal component analysis (PCA) was first performed as a dimensionality reduction to assess global trends in hematologic trajectories between survivors and non-survivors following TBI, specifically between scheduled and preterminal blood collections (Figure 2). Samples were categorized into biologically defined study periods based on visual analysis of CBC trajectories, including baseline (days -7, -4, -3, and -1), early suppression (days 1–8), nadir onset (days 10–13), critical/terminal period (days 14–22), early recovery (days 24–31), and late recovery (days 34–60). Centroids were added to visualize the average position of scheduled samples within each pre-defined study period and were also utilized to visualize the directional trends of each period over time. While both groups demonstrated highly aligned early hematological responses following irradiation, moving from the baseline to both early suppression and nadir onset phases, a stark divergence became apparent during the critical/terminal period. While survivors trend back towards baseline through the early and late recovery phases, non-survivors continue along an extended preterminal trajectory. The tight clustering of preterminal samples from non-survivors between the nadir and critical/terminal periods highlights a distinct late-stage hematologic phenotype associated with mortality. Although PCA is purely descriptive, this supports the patterns observed in the trendlines.

#### 2.1.3. Longitudinal Modeling Identifies Erythroid-Related Divergence Between Survivors and Non-Survivors

**Figure 3:**
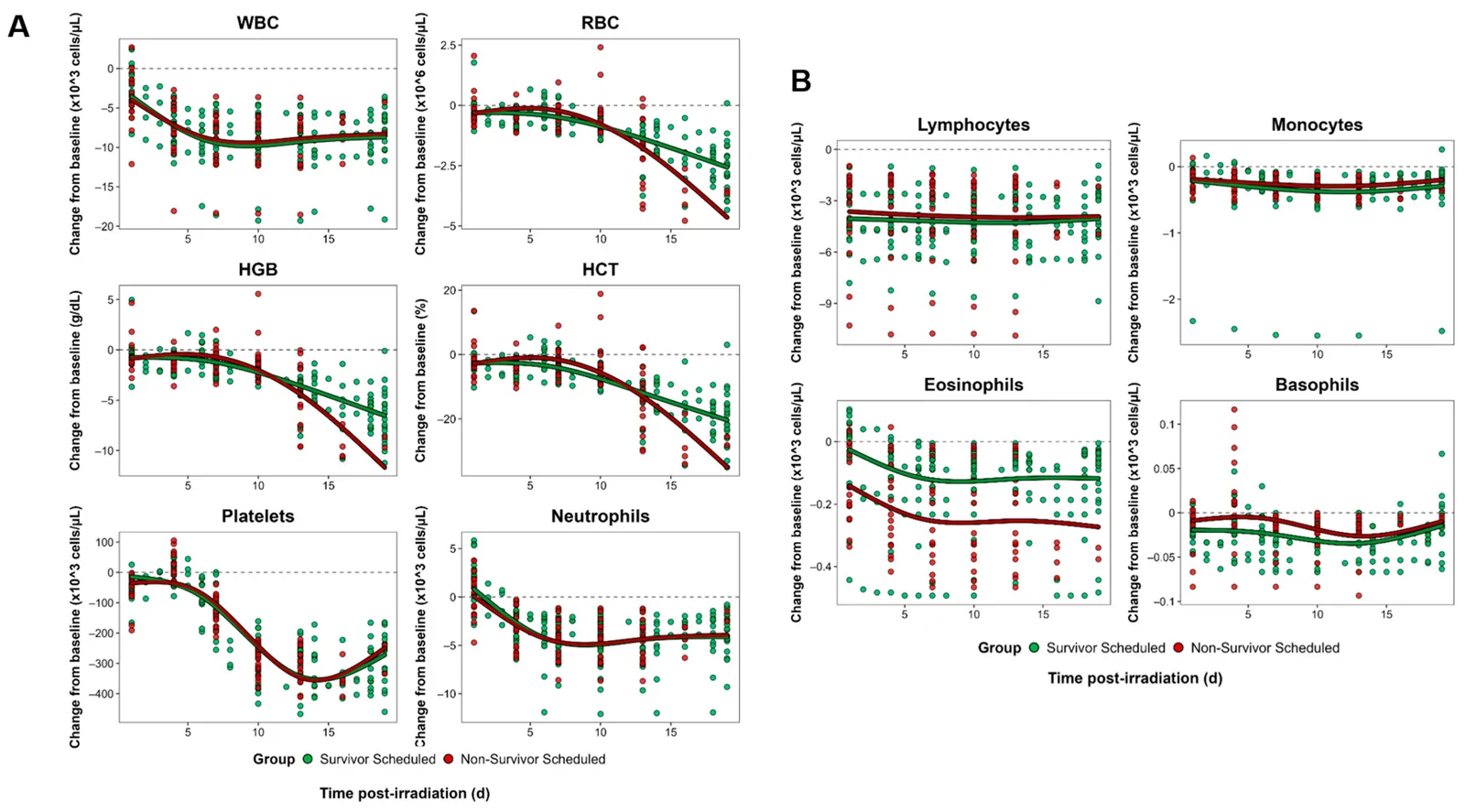
Survival-associated differences in survivors and non-survivors using scheduled blood cell samples after total-body irradiation between days 1–19. (**A**) White blood cell (WBC), red blood cell (RBC), hemoglobin (HGB), hematocrit (HCT), platelet, and neutrophil trajectories. (**B**) Lymphocyte, monocyte, eosinophil, and basophil trajectories.

Longitudinal modeling was performed using scheduled post-irradiation samples in survivors and non-survivors to determine if any parameters followed significantly different trajectories between days 1–19 (Figure 3A,B). Preterminal samples were excluded from this analysis, as they represent a distinct biological state that could alter or exaggerate the trajectories. Changes from baseline were plotted and lines were fit using natural cubic splines to account for variable non-linear trajectories. Some parameters showed substantial overlaps between survivors and non-survivors, including WBC, platelets, neutrophils, lymphocytes, and monocytes. Eosinophils had smaller changes between the two cohorts, while basophils generally followed the opposite trend. Most notably, erythroid-associated trajectories (RBC, HGB, and HCT) demonstrated significant survivor-associated trajectory differences, suggesting that non-survivors experienced progressively greater decreases from baseline in these parameters.

#### 2.1.4. Temporal Mapping of Hematopoietic Nadirs Identifies a Critical Window Associated with Mortality Following Total-Body Irradiation

To further define the precise temporal boundaries of post-irradiation hematologic suppression and recovery, the onset (“Nadir First”) and resolution (“Nadir Last”) of cell counts at their lowest values were quantified for each blood lineage and stratified by survival status (Supplementary Figure S2A,B). The clearest separation between survivors and non-survivors was observed within erythroid-associated parameters, including RBCs, HGB, and HCT. Survivors generally reached their nadirs later, approximately 21 days post-irradiation, whereas non-survivors exhibited earlier nadirs clustered more closely between days 14–18. This aligns with when non-survivors started to experience mortality.

Among highly radiosensitive cell types, such as WBCs, platelets, neutrophils, and lymphocytes, survivors exhibited highly compressed nadir profiles in which the last day of their nadir closely tracked to their first day. Although nadirs among WBCs, platelets, neutrophils, and lymphocytes occurred within a relatively similar time frame between survivors and non-survivors, important differences in nadir dynamics were still apparent. Non-survivors exhibited slightly delayed nadir values and slightly broader nadir distributions within these cell types. These nadirs also clustered within the critical period window post-irradiation, aligning closely with the majority of preterminal samples.

#### 2.1.5. Preterminal Samples Deviate from the Scheduled Non-Survivor Trajectory

An additional model was fit to determine whether preterminal samples represented a distinct hematologic state from non-survivors within the critical period from days 10–22 (Supplementary Table S2). CBC values were modeled as a change from each animal’s baseline, and a mixed effects model with natural splines was applied to determine whether preterminal samples deviated significantly from the expected scheduled non-survival trajectory. Animal ID was included as a random intercept to account for repeated measurements. When modeled from days 10–22 to include all preterminal samples, RBCs, HGB, and HCT were significantly decreased in preterminal samples when compared with scheduled non-survivor samples. A sensitivity analysis was then performed to model days 10–19 to determine whether the model was influenced by the two later preterminal samples at day 22 (Supplementary Table S3). In the day 10–19 window, RBCs, HGB, and HCT remained significant, but WBCs, lymphocytes, and monocytes were significantly lower. These results suggest that erythroid-related decline was the most robust marker of the preterminal state, with leukocyte suppression evident in the earlier day 10–19 window.

**Figure 4:**
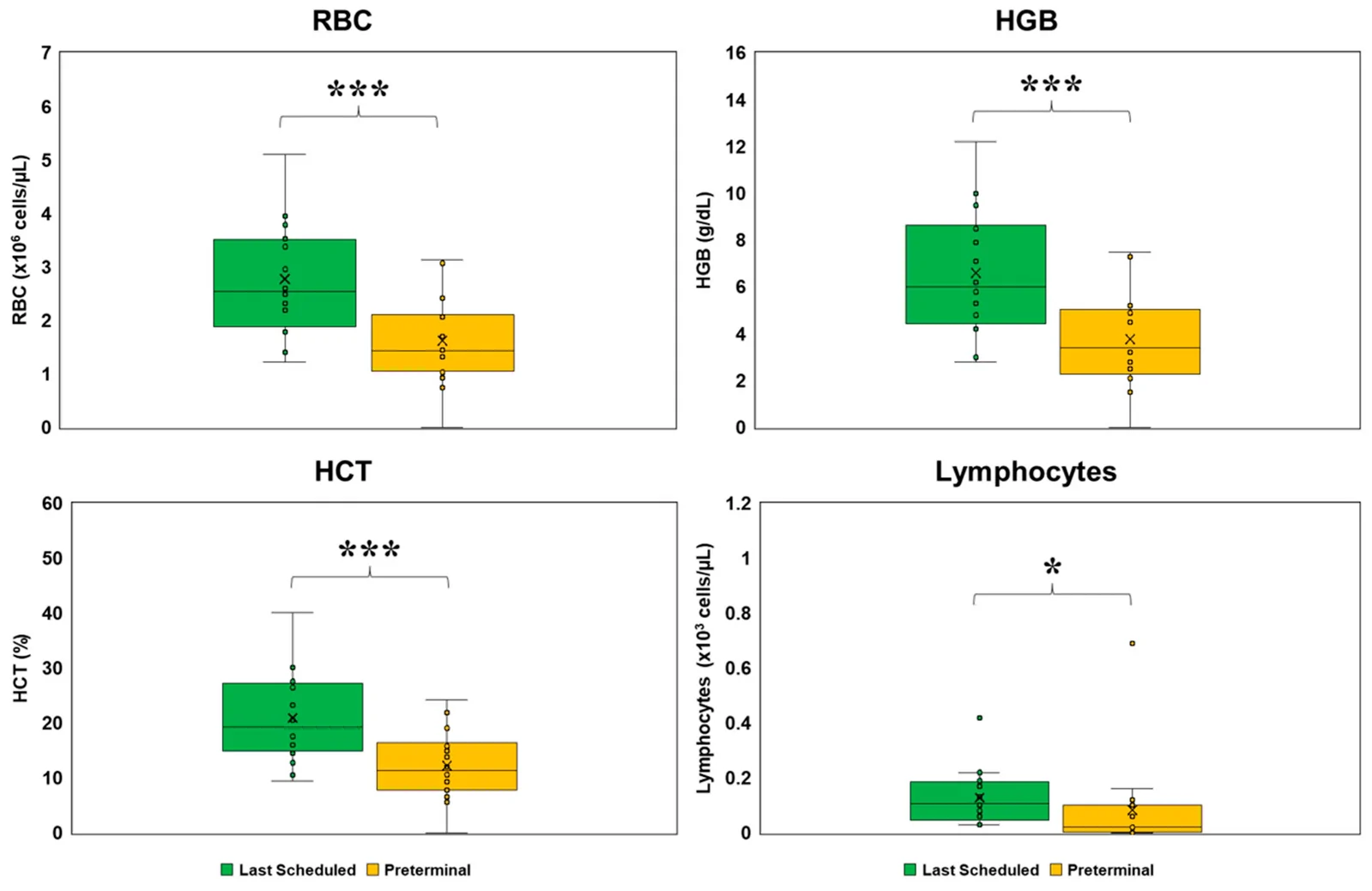
Boxplots comparing complete blood cell (CBC) parameters for the last scheduled timepoint to the preterminal timepoint in non-survivors. A Wilcoxon signed-rank test was used to assess within-subject differences, highlighting significant hematologic declines immediately preceding mortality. Statistical significance is denoted as *p* < 0.05 (*) and *p* < 0.001 (***). No significant differences were found in WBCs, platelets, neutrophils, monocytes, eosinophils, and basophils.

Pairwise comparisons were then performed between the last scheduled and preterminal samples in non-survivors as a supportive within animal analysis (Figure 4). Consistent with the time-adjusted preterminal model, marked terminal suppression in erythroid parameters was evident, with RBCs, HGB, and HCT all showing significant decreases (*p* < 0.001) prior to death. Lymphocyte counts also experienced a statistically significant depletion (*p* < 0.05). Together, these sharp reductions illustrate that terminal failure leading up to mortality may be characterized by acute erythroid, as noted earlier, and lymphocytic depletion. Other CBC parameters, such as WBCs, platelets, neutrophils, monocytes, eosinophils, and basophils, did not differ significantly in the pairwise non-survivor scheduled vs. preterminal sample comparisons. This sensitivity model provided some insight into time-specific changes, particularly in WBCs, lymphocytes, and monocytes, that were not evident when including the two preterminal samples of animals euthanized on day 22.

### 2.2. Serum Biochemistry Response to TBI

#### 2.2.1. Longitudinal Serum Chemistry Profiles Demonstrate Late Disruption as Non-Survivors Approached the Preterminal State

**Figure 5:**
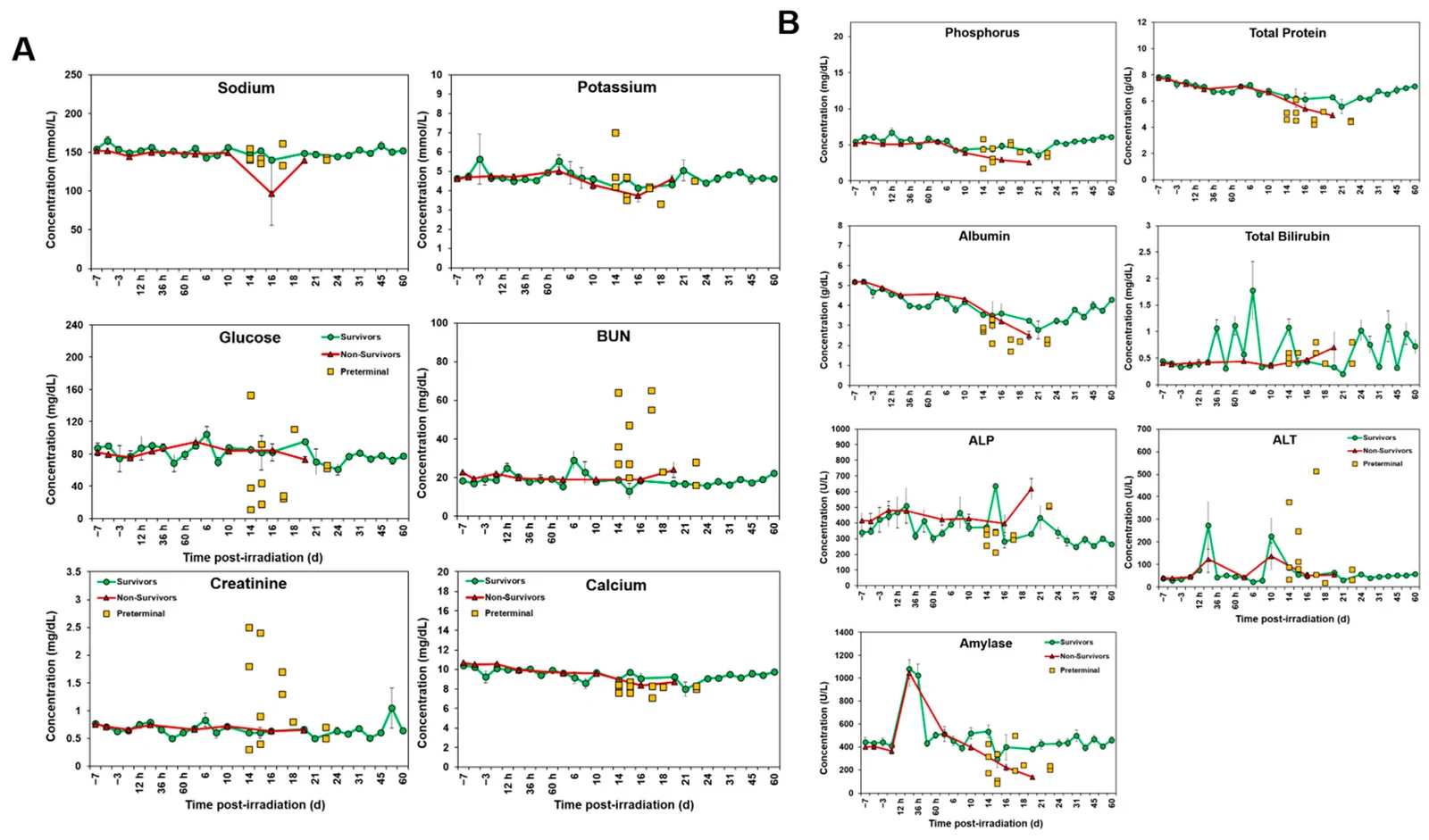
Raw chemistry data stratified by survivors, non-survivors, and preterminal data. These metabolic parameters capture serum biochemical responses within sodium, potassium, glucose, blood urea nitrogen (BUN), creatinine, and calcium (**A**), and phosphorus, total protein, albumin, total bilirubin, alkaline phosphatase (ALP), alanine aminotransferase (ALT), and amylase (**B**), preceding mortality and compare recovery trajectories between outcome groups.

Serum chemistry analyses revealed time-dependent biochemical trends between survivors, non-survivors, and preterminal collections (Figure 5A,B). While several biomarkers, such as sodium, calcium, and potassium, remained relatively stable during the early post-exposure phase, the critical period (primarily between days 14–18) and the preterminal phase were characterized by greater biochemical disruption in non-survivors.

Renal parameters remained relatively stable among survivors, in contrast to the acute deviations seen in non-survivors immediately preceding mortality. Several individual preterminal samples between days 14–17 revealed marked increases in BUN and creatinine. Phosphorus generally decreased in non-survivors, but preterminal samples did not show a consistent trend. Sodium concentrations were tightly regulated in both groups, except for a notable decrease in non-survivors on day 16. Potassium showed minor fluctuations over the course of the study period in both cohorts, with a gradual decline beginning around day 10.

Protein-related parameters also showed significant changes post-irradiation. Both groups experienced a steady decline in total protein and albumin from baseline through the first two weeks post-irradiation. Surviving animals demonstrated gradual recovery toward baseline by day 60, while non-survivors generally exhibited persistent reductions in total protein and albumin prior to mortality or preterminal collection, with some individual preterminal samples demonstrating severe hypoproteinemia and hypoalbuminemia. ALT demonstrated variable trajectories, with early elevations observed in both cohorts and a more exaggerated increase in some preterminal samples. ALP also displayed variable trends between cohorts, with occasional elevations in scheduled samples but no consistent preterminal pattern. Total bilirubin also fluctuated throughout the study, with more pronounced variability in survivors and some modest increases in preterminal samples.

Pancreatic function and glycemic regulation also revealed major alterations over time. Amylase concentrations showed a hyperacute spike 12–36 h post-irradiation in both cohorts, after which amylase levels generally stabilized in survivors but underwent a progressive decline in non-survivors from day 4 onward. Glucose concentrations fluctuated moderately in both cohorts throughout the study, with several preterminal samples demonstrating marked dispersion.

#### 2.2.2. PCA Visualization Supports Separation of the Preterminal State in Serum Biochemistry

**Figure 6:**
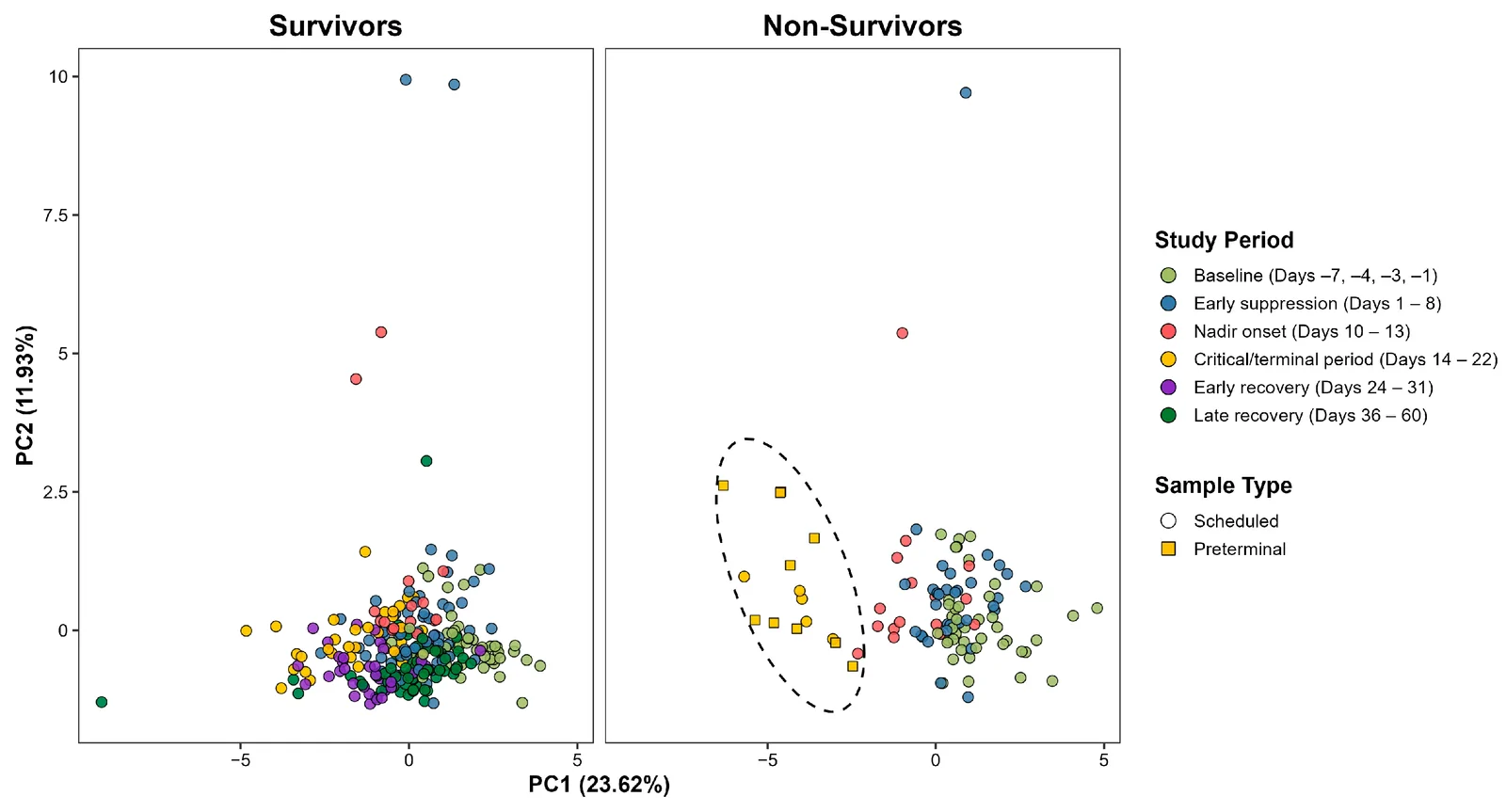
Principal component analysis (PCA) of serum chemistry parameters. Between survivors and non-survivors, biochemical responses from scheduled and preterminal blood collections at various pre- and post-irradiation windows were analyzed to evaluate multivariate distribution patterns across the combined effects of total-body exposures.

Unsupervised PCA was performed on serum chemistry parameters to evaluate global biochemical trajectories between survivors and non-survivors following TBI (Figure 6). It is important to note that the limited chemistry collection time points reduced the ability to view temporal resolution between sequential study periods. In survivors, chemistry parameters remained tightly clustered and heavily overlapped with each other across all longitudinal study periods, from baseline to late recovery. On the other hand, non-surviving animals demonstrated a stark divergence from this central homeostatic cluster, specifically during the critical/terminal period, which is similar to trends observed in the CBC PCA. This shift is characterized by a distinct leftward migration along the PC1 axis away from the baseline and early suppression states. Preterminal samples from these non-surviving animals clustered tightly within this segregated space, which is denoted by the dashed ellipse.

#### 2.2.3. Serum Chemistry Dynamics and Timing of Preterminal Nadirs

For non-survivors, both the initial (“Nadir First”) and final (“Nadir Last”) nadirs across virtually all parameters were more temporally compressed compared with survivors (Supplementary Figure S3A,B). Consistent with the timeline established for CBC parameters, serum chemistry kinetics reached their respective nadirs during the critical period. In sharp contrast, surviving animals exhibited highly protracted and widely distributed temporal nadir trends. The extreme values for survivors were scattered across the entire 60-day post-irradiation study period, with many animals not reaching their final nadirs or physiological turning points until days 20–60. This wide-ranging window reflects a sustained period of physiological adaptation and recovery, ultimately culminating in the re-establishment of metabolic homeostasis and long-term survival.

#### 2.2.4. Longitudinal Modeling of Scheduled Serum Biochemistry Trajectories Did Not Identify Significant Survival-Associated Differences

Similar to the model applied to CBC data, longitudinal modeling changes from baseline in scheduled biochemistry samples between days 1–19 was performed. However, unlike CBC, this model did not reveal any significant differences in the trajectories between survivors and non-survivors in serum biochemistry parameters. Sodium, albumin, and phosphorus showed nominal significance, but these did not remain significant after false discovery rate (FDR) correction.

#### 2.2.5. Biochemical Decline Results in Preterminal Renal and Glycemic Changes

**Figure 7:**
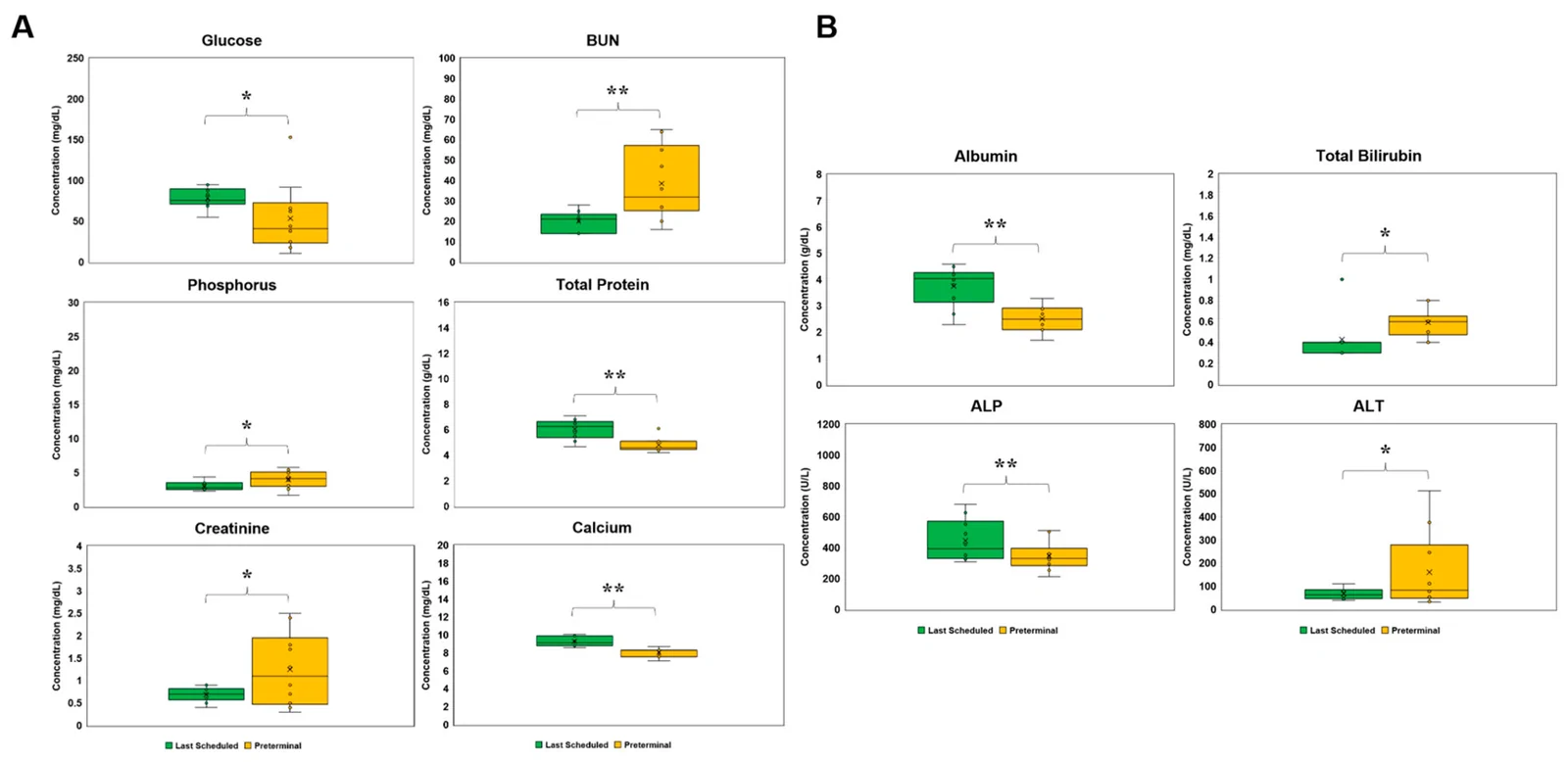
Boxplots comparing serum chemistry parameters for the last scheduled timepoint to the preterminal timepoint in non-survivors. A Wilcoxon signed-rank test was used to assess within-subject differences, highlighting significant biochemical changes immediately preceding mortality in Glucose, Blood Urea Nitrogen (BUN), Phosphorus, Total Protein, Creatinine, and Calcium (**A**), and Albumin, Total Bilirubin, Alkaline Phosphatase (ALP), and Alanine Aminotransferase (**B**). Statistical significance is denoted as *p* < 0.05 (*) and *p* < 0.01 (**). No significant differences were found in sodium, potassium, and amylase.

Due to the sparseness of both scheduled and preterminal chemistry samples within the critical period, the spline-based longitudinal model that determines if preterminal samples significantly deviated from the non-survivor trajectory was unable to be reliably fit. Therefore, within-subject comparisons were performed between the last scheduled timepoint to the subsequent preterminal collection timepoint in non-surviving animals (Figure 7A,B). These paired comparisons identified significant increases in BUN (*p* < 0.01), creatinine (*p* < 0.05), and phosphorus (*p* < 0.05), with several preterminal samples displaying outlying elevations. Concurrently, calcium levels experienced a significant drop (*p* < 0.01), further emphasizing a major breakdown in the body’s mineral balance.

Liver function, protein homeostasis, and glucose concentrations also demonstrated severe preterminal decreases. Total protein (*p* < 0.01), albumin (*p* < 0.01), and glucose (*p* < 0.05) levels dropped precipitously. Impaired liver function was apparent from significant increases in ALT (*p* < 0.05) and total bilirubin (*p* < 0.05). Interestingly, ALP activity decreased significantly (*p* < 0.01) during the preterminal phase, despite its broad late-stage elevations observed across the wider cohort. However, despite these prominent shifts, the absence of significant changes in sodium, potassium, and amylase suggests that these biomarkers were less consistently associated with the preterminal state.

## 3. Discussion

Biomarkers provide a critical map of the body’s response to ionizing radiation by quantifying discrete biological shifts that precede physiological decline. The well-characterized kinetics of hematologic parameters following ionizing radiation exposure have established CBCs as a cornerstone for assessing radiation-induced injury [5,28,29,30,43]. These indicators serve as vital surrogate endpoints that bridge the gap between initial molecular damage and symptomatic outcomes, even when a patient appears clinically stable. Because ionizing radiation disrupts the rapid turnover of hematopoietic stem and progenitor cells, peripheral blood counts give a real-time narrative of bone marrow health, providing a final window at the preterminal stage for aggressive supportive care or experimental therapies before multi-organ failure clinically manifests and becomes potentially untreatable. When paired with serum chemistry data, these complementary analyses enable researchers and clinicians to more accurately identify homeostatic decline and inform supportive care decisions.

Extensive work on preterminal biomarkers of H-ARS has been conducted, focusing on defining proteomic, metabolomic, and transcriptomic patterns of injury in whole blood, serum, and plasma samples of irradiated NHPs exposed to total-body ^60^Co γ-radiation. In plasma from NHPs exposed to total-body radiation, liquid chromatography–tandem mass spectrometry analysis identified correlations between preterminal status and proteins related to hemostasis and inflammation, including integrin alpha and thrombospondin [20]. Consistent with these findings, mass spectrometry-based proteomics revealed pronounced dysregulation of immune response and hemostasis pathways in serum samples collected from NHPs exposed to 7.2 or 7.6 Gy total-body radiation, specifically those associated with platelet function [21]. Metabolomic studies have also implicated the glycerophospholipid metabolism, steroid hormone biosynthesis, and sphingolipid metabolism pathways as pathways associated with lethality and preterminal decline after 7.2 or 7.6 Gy total-body radiation exposure [18,19]. Differential mobility spectrometry-mass spectrometry (DMS-MS) identified trimethyl-L-lysine (TML) and hypoxanthine as preterminal urinary radiation biomarkers seven days following 10 Gy TBI after strong cation exchange-solid phase extraction (SCX-SPE) preparation, with hypoxanthine demonstrating translational relevance across both humans and NHPs [44]. Additionally, transcriptomic analyses in lethally irradiated rhesus macaques demonstrated widespread downregulation of radiation-responsive genes during the preterminal stage following 7 Gy TBI, suggesting that while gene expression profiles may lose utility for biodosimetry at late stages post-exposure, they may serve as indicators of emerging preterminal decline [22]. Collectively, these studies demonstrate that the preterminal state is characterized by systemic decline across immune, hemostatic, metabolic, and transcriptional pathways.

Although omics studies provide invaluable biological insight, these sophisticated analyses require access to high-resolution instrumentation, specialized analytical pipelines, and rigorous methodological standardization to ensure reproducibility and translational validity. The goal of the current investigation, however, was to determine whether this preterminal state could be captured using routinely deployable clinical assays, such as CBCs and serum chemistry. In the CBC analysis, the post-irradiation period was dictated by a critical window between days 14–22, during which survivors began to stabilize or recover, while non-survivors showed progressive hematological and biochemical deterioration. PCA further supported this pattern, showing an extended critical/terminal period that diverged from recovery in non-survivors that was not present in survivors.

A key finding from this analysis was the divergence of leukocyte and erythrocyte lineages. Radiation-sensitive populations, including WBCs, neutrophils, and platelets, demonstrated rapid and near-complete depletion within the first 10 days post-exposure across all animals, regardless of survival outcome. When evaluating the temporal boundaries of these parameters, survivors reached their nadir earlier and experienced a rapid cellular recovery thereafter, whereas non-survivors exhibited delayed nadir values and broader nadir distributions. Because these short-lived cells were depleted uniformly as a baseline response to TBI, their early suppression served as a highly sensitive indicator of radiation exposure but lacked the prognostic specificity required to differentiate by survival status. Conversely, the erythroid lineage (RBC, HGB, and HCT) demonstrated clear late divergence between survivors and non-survivors that was robust across both the primary and sensitivity preterminal models, indicating that this result was not driven by the two later preterminal samples at day 22. The sensitivity preterminal analysis demonstrated that significant reductions in WBCs, lymphocytes, and monocytes were present in the day 10–19 window, but were not consistently significant when including the two later preterminal samples. While surviving animals successfully stabilized their baseline values and initiated recovery, non-survivors demonstrated a truncated trajectory resulting in hematopoietic failure.

Serum chemistry profiles complemented the hematologic findings by providing evidence of greater systemic imbalances, although scheduled chemistry trajectories were less discriminatory than hematologic trajectories. Nearly a week before clinical collapse became apparent, animals destined for mortality exhibited downward trends in sodium, phosphorus, total protein, and amylase concentrations. However, these scheduled trajectories were not associated with non-survivor status after FDR correction in the longitudinal spline-based modeling. Many of the parameters followed similar trajectories across cohorts. However, preterminal serum biochemistry samples greatly varied in response. When comparing last scheduled samples to preterminal samples in non-survivors, many parameters, including glucose, BUN, phosphorus, total protein, creatinine, calcium, albumin, total bilirubin, ALP, and ALT, showed significant changes immediately prior to death. These findings suggest that the greatest abnormalities were observed in preterminal samples rather than as scheduled trajectory differences.

Together, the preterminal samples for CBC and serum chemistry capture a synchronized, multi-system breakdown where hematologic failure potentially contributes to metabolic and biochemical collapse. This phenotype was most apparent during days 14–22 post-irradiation, when non-survivors demonstrated progressive hematologic decline accompanied by renal and hepatic alterations, as well as protein, glucose, and calcium imbalances. One plausible interpretation is that declining RBC, HGB, and HCT concentrations may contribute to reduced oxygen-carrying capacity and broader systemic dysregulation at the preterminal state. In parallel, increases in total bilirubin are suggestive of altered radiation-induced heme handling. Recent studies have implicated ferroptosis and tissue iron deposition as contributors to delayed recovery post-irradiation [45], suggesting that disrupted erythroid activity and iron handling may be important characterizations of the preterminal phenotype. Additionally, the simultaneous and sharp drops in both RBCs and circulating proteins right before death suggest possible radiation-induced endothelial leakage, a phenomenon where cellular elements and macromolecular proteins escape the vasculature together [46]. The sudden breakdown in endothelial barrier function was mechanistically confirmed by the concurrent crash in calcium, which binds heavily to circulating albumin and is pulled out of the bloodstream as plasma proteins leak into the interstitial space [46]. However, these suggested mechanisms cannot be validated directly in this study and are purely speculative.

By mapping these temporal trajectories, this study suggests that the transition from H-ARS to lethality is not a prolonged, uniform decline, but rather a rapid and highly synchronized multi-system deterioration clustered within a critical post-exposure window. Recognizing this timeline provides a vital framework for countermeasure development, identifying days 14–22 post-irradiation as a narrow yet crucial therapeutic window for supportive care. Characterizing these preterminal biomarkers of H-ARS further identifies what specific blood cell and biochemical targets are at the greatest risk of additional injury in the preterminal state.

A few limitations were present in this study that should be acknowledged to improve the scope of future investigations. Animals exposed to two doses were pooled because both doses fall within the LD_60–80/60_ range and the primary objective was to characterize hematological and biochemical response associated with survival and the preterminal state rather than to characterize radiation dose-dependent responses. Although pooling increased the sample size and available preterminal samples, dose-dependent differences were not directly evaluated and may have contributed to variability in the observed responses. Notably, serum chemistry collections were less abundant than CBC collections, which limited the model-based analysis of preterminal biochemistry deviations. Lastly, additional studies should be performed with tissue-specific investigations to further support our findings here. Despite these limitations, this study demonstrated that routine CBC and serum biochemistry collections can help define the preterminal phenotype following high doses of total-body ionizing radiation.

## 4. Materials and Methods

### 4.1. Experimental Design

This study used male and female rhesus macaques to identify blood-based biomarkers that distinguish the preterminal state from earlier stages of radiation injury. A total of 28 survivors and 16 non-survivors were included in this analysis. The objective was to characterize the CBC and serum chemistry profiles in preterminal samples collected immediately prior to euthanasia due to moribund conditions and compare their values with samples from survivors and non-survivors at scheduled timepoints prior to moribundity. Animals were exposed to 7.2 or 7.4 total-body ^60^Co γ-radiation, which were in the range of LD_60–80/60_ for rhesus macaques.

Samples from both cohorts were pooled because the goal of this investigation was to profile and elucidate CBC and serum chemistry patterns associated with survival outcome and preterminal decline. Although preterminal samples were available for animals from both the cohorts for CBC data (*n* = 16), preterminal samples were only available for the 7.4 Gy cohort for the serum biochemistry data (*n* = 10). As a result, preterminal serum chemistry findings were interpreted with these limitations in mind.

Survival outcomes, hematopoietic responses, and biochemical perturbations were monitored for 60 days post-irradiation using CBC and serum chemistry analyses at scheduled timepoints. Two types of collections were performed in this study: scheduled and non-scheduled. Survivors have only scheduled collections, while non-survivors have both scheduled and non-scheduled collections. Non-scheduled collections in non-survivors represent preterminal samples, or samples collected immediately prior to euthanasia in moribund animals. These preterminal samples were collected throughout the critical period, or the period between days 10 to 20 during which terminal decline becomes more likely to manifest. In this study, two animals were euthanized on day 22, so we have expanded this window for additional screening to days 10–22 to accurately assess all preterminal samples.

### 4.2. Animals

This study included forty-four naïve rhesus macaques (*Macaca mulatta*) procured from Worldwide Primate, Inc. (Miami, FL, USA) and the National Institutes of Health Animal Center (NIHAC, Poolesville, MD, USA). Upon arrival, each animal entered a seven-week minimum quarantine period with thorough health assessments conducted to confirm experimental suitability. Veterinary screening confirmed the absence of key pathogens such as herpes B virus (*Macacine herpesvirus* 1), simian T-cell leukemia virus type 1 (STLV-1), simian immunodeficiency virus (SIV), and simian retrovirus (SRV) types 1, 2, 3, and 5, as well as gastrointestinal bacterial agents (Salmonella, Shigella, Klebsiella, Yersinia) and simian varicella virus. Immunization status for measles was verified through vaccination or antibody titer assessment. Final study inclusion was determined by veterinary evaluation of health records. The experimental cohort included healthy animals ranging in age from 2.8–6.3 years and weighing 3.6–7.01 kg.

Prior to group assignment, animals were stratified by sex and quarantine weight variation. Housing occurred in individual cages within an Association for Assessment and Accreditation of Laboratory Animal Care (AAALAC) International-accredited vivarium. Environmental conditions included temperature control at 22 ± 2 °C, relative humidity at 30–70%, air exchange at 10–15 cycles per hour, and 12 h light/dark cycles. All animals received appropriate primate diets twice daily with water *ad libitum*. Social contact was maintained through visual, auditory, and tactile interactions with adjacent animals. Daily health monitoring, environmental enrichment, and husbandry followed established protocols as described previously [47]. All procedures received approval from the IACUC of the Armed Forces Radiobiology Research Institute (AFRRI), Uniformed Services University of the Health Sciences (USUHS), and the Department of War Animal Care and Use Review Office (ACURO). Studies followed the *Guide for the Care and Use of Laboratory Animals* and ARRIVE (Animal Research: Reporting of In Vivo Experiments) guidelines [48].

### 4.3. Irradiation

Irradiation was performed with up to eight animals on any specific day. Food was withheld for 12–18 h before exposure to minimize radiation-induced emesis while water remained freely available. Intramuscular (*im*) ketamine (10–15 mg/kg) was administered 30–40 min prior to irradiation. Abdominal width measurements using digital calipers (iGaging, San Clemente, CA, USA) enabled precise individual dose calculation. Animals received total-body ^60^Co γ-radiation delivered bilaterally at 0.6 Gy/min. Two animals were irradiated simultaneously, positioned in separate Plexiglas containers with opposing orientations to ensure uniform exposure distribution. Irradiation targeted the abdominal core from both sides to achieve total-body midline dosing conducted at the high-level ^60^Co facility (HLCF) at AFRRI. Dosimetric protocols and irradiation methodology for rhesus macaques have been discussed earlier [47]. Radiation field uniformity was maintained within ±1.5% variation at animal positioning sites. Primary dosimetric measurements used alanine/electron paramagnetic resonance (EPR) as previously described [47]. Cylindrical water phantoms (34.5 cm length) with outer diameters of 5.08, 6.99, 10.0, 12.5, and 15.1 cm were employed for dose rate validation. Each phantom underwent four dose rate measurements with relative standard deviation below 0.4%. Continuous video monitoring ensured animal safety throughout the irradiation procedure. Following irradiation, animals were returned to the vivarium for observation until full anesthetic recovery and normal mobility restoration.

### 4.4. Supportive Care and Clinical Evaluation

Post-irradiation monitoring was employed for 60 days with survival as the primary outcome. Health assessments at the cage side occurred twice daily by either husbandry staff or research personnel to identify signs of distress or physical complications. Monitoring frequency increased to three times daily during days 10–20 post-irradiation, referred to as the “critical period,” with approximately 8–10 h intervals between observations due to heightened morbidity risk. Notably, two moribund animals were euthanized outside of this window at day 22. Supportive care decisions incorporated multiple assessment parameters, including hematological monitoring through CBC analysis, behavioral evaluations through cage-side observations, and physical examinations to assess overall condition and detect clinical signs of radiation illness. This comprehensive approach ensured all clinical interventions were data-driven and individualized to each animal’s health status. Detailed observation protocols have been described in previous publications [19,47]. Treatment interventions included fluid replacement therapy, alternative antimicrobial agents, antipyretics, antidiarrheal agents, analgesics, antiemetics, mucosal ulcer treatment, and dietary supplementation [47]. Blood transfusions were not used during these studies.

### 4.5. Euthanasia

Euthanasia procedures followed the *Guide for the Care and Use of Laboratory Animals*, approved IACUC protocols, and the American Veterinary Medical Association’s (AVMA) Guidelines for Animal Euthanasia [48,49]. Animals underwent euthanasia upon reaching moribund status, defined as irreversible clinical deterioration. Moribundity served as a mortality endpoint to prevent unnecessary pain and suffering [47]. Moribundity determination used comprehensive assessment criteria incorporating clinical observations and objective measurements. Animals were classified as moribund when exhibiting multiple severe clinical or laboratory conditions, including significant weight loss, anorexia, inability to feed or drink, decreased responsiveness, abnormal body temperature, and severe anemia or thrombocytopenia. The complete criteria for moribundity are provided in the Supplementary Information, which has specific thresholds for hematological and serum biochemical parameters. In brief, an animal was considered moribund when it met the predefined clinical and/or laboratory criteria, including the specified CBC and chemistry thresholds detailed in the Supplementary Information. Veterinary evaluation of the respiratory, gastrointestinal, urogenital, nervous, and integumentary systems, along with hematologic and serum biochemical findings, was considered collectively for the final determination of moribundity. No single clinical or laboratory criterion alone warranted euthanasia. Euthanasia decisions involved collaborative consultation among veterinary and research staff members. Decisions were never based solely on clinical symptoms and required veterinary consultation for all determinations. Moribund animals first received *im* ketamine sedation (5–15 mg/kg) followed by isoflurane maintenance (1–5%; Baxter Healthcare Corporation, Deerfield, IL, USA) administered through face mask with oxygen delivery (1–4 L per minute). Euthanasia was conducted using intravenous pentobarbital sodium (100 mg/kg, 1–5 mL; Virbac AH Inc., Fort Worth, TX, USA) delivered through either the saphenous or cephalic veins using a 20–25-gauge needle. When peripheral venous access was not feasible, pentobarbital was administered via intracardiac injection. Death confirmation was accomplished through cardiac auscultation and pulse palpation.

### 4.6. Blood Sample Collection

Venipuncture procedures were performed using saphenous veins (lower extremity) or brachial veins (upper extremity) approximately 1–3 h following feeding. Prior to blood collection, animals underwent several weeks of conditioning to acclimate them to restraint chairs. They received treats before, during, and after training sessions to provide positive reinforcement and reward behavior. All collections were conducted on fully conscious animals positioned in restraint chairs using the pole-and-collar method to minimize movement. Collection procedures involved applying a tourniquet followed by disinfecting the puncture site with 70% isopropyl alcohol wipes to maintain aseptic conditions and improve vein visibility. Blood sampling utilized 3 mL disposable luer-lock syringes with 22–25-gauge needles, depending on animal weight. Following collection, pressure was applied with sterile gauze to facilitate hemostasis and prevent hematoma formation.

Sample collection schedules differed by analysis type and radiation dose. CBC samples were obtained from both cohorts from pre-irradiation days -7, -4, -3, and -1, and post-irradiation days 1–8, 10, 12–22, 24–26, 28, 30, 31, 34, 38, 42, 45, 50, 52, and 60. Chemistry analysis utilized samples the 7.4 Gy cohort only, with samples collected on pre-irradiation days -7, -4, -3, -1, and post-irradiation at 12 h, day 1, 36 h, day 2, 60 h, and days 4, 6, 7, 10, 14–16, 19, 21, 24, 28, 31, 38, 45, 50, and 60. Final collection endpoints varied by survival outcome, with survivor collections continued through day 60, non-survivor scheduled collections between days 10–19, and preterminal samples obtained between days 14–22 based on individual animal condition and moribundity determination status.

### 4.7. CBC Analysis

Blood samples collected for CBC analysis were obtained in ethylenediaminetetraacetic acid (EDTA) tubes (Sarstedt Inc., Newton, NC, USA) and analyzed using a Bayer Advia-120 Hematology Analyzer (Siemens Medical Solutions, Malvern, PA, USA). CBC analysis was conducted to assess ten hematological parameters, including WBCs, RBCs, HCT, HGB, and absolute counts for platelets, neutrophils, lymphocytes, monocytes, eosinophils, and basophils.

### 4.8. Serum Chemistry Analysis

Serum chemistry samples were collected in serum-separator tubes and allowed to clot for a minimum 30 min period at room temperature before centrifugation (10 min at 400× *g*). The resulting serum was separated and then stored at −70 °C until analysis. Chemistry analysis was performed using a Vitros 350 Automatic Biochemistry Analyzer (Ortho Clinical Diagnostics, Markham, ON, Canada). Thirteen parameters were assessed, including sodium, potassium, glucose, BUN, creatinine, calcium, phosphorus, total protein, albumin, total bilirubin, ALP, ALT, and amylase.

### 4.9. Statistical Analysis

The goal of this study was to determine whether survivors and non-survivors showed different hematologic and serum chemistry trajectories following irradiation, and whether preterminal samples represented a distinct biological state among non-survivors. Because non-survivors stopped contributing data after euthanasia, analyses were performed within defined windows to reduce survivor-related missingness.

Unsupervised PCA was performed as an exploratory visualization to evaluate global patterns in raw hematologic and chemistry profiles separately over time among samples collected from scheduled survivors, scheduled non-survivors, and preterminal non-survivors. Prior to PCA, each parameter was z-scored by centering to a mean of 0 and scaling to unit variance to ensure all parameters contributed equally despite being on different scales. Samples were grouped into biologically defined study periods including baseline, early suppression, nadir onset, critical/terminal period, early recovery, and late recovery by visually evaluating trendlines. PCA was used only to explore global trends in CBC and chemistry data, while pairwise comparisons and longitudinal modeling were performed for statistical rigor.

Pairwise Wilcoxon signed-rank tests were performed to assess significant differences between last scheduled samples and preterminal samples in non-survivors to determine if an additional change occurred as the animal approached the preterminal state. It is important to note that the interval between the last scheduled and preterminal collection varied across animals, and these comparisons do not account for any time-dependent changes.

Longitudinal CBC trajectories were analyzed using regression models with natural cubic splines by study day to account for non-linear patterns of depletion and recovery following irradiation. Each CBC value was expressed as a change from each animal’s own baseline. For each animal and parameter, a baseline was calculated as the means of pre-irradiation samples. Two separate mixed effect models with natural splines were fit. The first model analyzed the post-irradiation trajectories between survivors and non-survivors from days 1–19 (Supplementary Table S1). Preterminal samples were excluded from this model. A second model was fit to determine whether preterminal samples deviated from the expected scheduled non-survivor trajectory during the critical period from days 10–22 (Supplementary Table S2). An additional model was fit as a sensitivity analysis between days 10–19 to determine if the two preterminal samples at day 22, which seemed to show some degree of recovery, influenced the results (Supplementary Table S3). Notably, longitudinal modeling was performed for serum biochemistry data to compare survivors to non-survivors within the common timepoint window, but no parameters remained significant after FDR correction. Additionally, due to the minimal amount of preterminal samples available for serum biochemistry, the second model was unable to be performed reliably. *p*-values were adjusted across parameters using the Benjamini–Hochberg FDR procedure. Results were considered significant at FDR < 0.05.

## Data Availability

All relevant data are within the manuscript.

## Supplementary Materials

The following supporting information can be downloaded at https://www.mdpi.com/article/10.3390/ijms27177709/s1.
