# Supplementary material for: Blood-Based Biomarkers for the Preterminal Stage Using Rhesus Macaques Exposed to Acute Total-Body Radiation
Source: Int J Mol Sci. 2026 Aug 28;27(17):7709. doi: 10.3390/ijms27177709 (PMC13565886; doi:10.3390/ijms27177709)
Supplement: Supplementary file 1 [file ijms-27-07709-s001.zip › 2026-08-20 Supplementary Information.pdf]

Supplementary Information: Euthanasia Criteria

To minimize pain and distress, moribundity served as a scientific surrogate for mortality to guide humane endpoints and prompt euthanasia. In this study, NHPs were evaluated against a comprehensive set of clinical parameters to assess moribundity: a 10% weight loss from baseline (monitored during routine blood collection chair restraints), complete anorexia for two consecutive days accompanied by physical deterioration, persistent weakness or reluctance to stand exceeding one hour, and minimal or absent responsiveness to external stimuli. Physiological criteria include core body temperature dropping below 96.6 °F following a period of febrile neutropenia (body temperature over 103 °F with fewer than 500 neutrophils/µL), severe anemia (hematocrit under 13% due to acute blood loss or hemoglobin under 40 g/L), and severe thrombocytopenia (fewer than 10,000 platelets/µL). Additionally, indicators of severe organ dysfunction with a poor prognosis warrant evaluation, including respiratory distress (dyspnea or severe cyanosis); life-threatening gastrointestinal events such as hemorrhagic diarrhea, obstruction, or peritonitis; urogenital decline indicated by renal failure parameters; central nervous system signs including sustained depression, seizures, or paralysis; and integumentary breakdown such as non-healing wounds or repeated self-trauma.

Determining a state of moribundity relies on a collaborative, team-based assessment involving the Principal Investigator, institutional veterinarians, veterinary technicians, research personnel, and husbandry staff. No single parameter automatically triggers euthanasia without explicit clinical veterinary recommendation, nor is there a fixed combination of criteria that replaces veterinary consultation. To ensure close oversight during high-risk/critical periods, research and veterinary personnel increased monitoring to every 8 to 10 hours between days 11 and 20 post-irradiation. Once a consensus confirms that an animal has reached an irreversible moribund state, humane euthanasia is promptly administered in consultation with Department of Laboratory Animal Resources (DLAR) veterinarians.
